# Abscess in the posterior region of the uterus due to *Streptococcus thoraltensis* in 38‑year‑old female: A case report

**DOI:** 10.3892/mi.2025.215

**Published:** 2025-01-13

**Authors:** Ingrid Janette Gónzalez Arceo, Gabriela Andrea Robles Rojo

**Affiliations:** Faculty of Medicine, University of Colima, 28040 Colima, Mexico

**Keywords:** abscess, uterus, *Streptococcus thoraltensis*, uncommon pathogen

## Abstract

*Streptococcus thoraltensis* (*S. thoraltensis*) is a bacterium usually present in the gut microbiome of quadruped mammals. *S. thoraltensis* is not considered pathogenic for humans; however, several reports have identified it as the etiological agent in cases of chorioamnionitis, postpartum pneumonia and fever of unknown origin. Furthermore, it has been isolated in samples from patients with endocarditis both with and without heart valve replacement. The present study describes the case of a 38-year-old healthy female patient who experienced acute abdominal pain accompanied by dysuria, vesical tenesmus and constipation. A computed tomography scan revealed a retro-uterine cystic mass due to a bacterial abscess. Following surgical drainage, microbiological culture identified *S. thoraltensis* as the etiological agent. The patient was thus treated with doxycycline and metronidazole, and exhibited a successful response to treatment. The increasing occurrence of *S. thoraltensis* in human infections suggests potential changes in the epidemiological profile of this bacterium. It is possible that human activity contributes directly or indirectly to the emergence of new pathogens.

## Introduction

*Streptococcus thoraltensis* (*S. thoraltensis*) is an alpha-hemolytic nonmotile, anaerobic, non-sporulating, Gram-negative *Streptococcus*. It was initially discovered in 1997(1). It commonly inhabits the urogenital and gastrointestinal tracts of pigs and rabbits (2) and constitutes a component of the normal gut microbiota of quadruped mammals (3). The overuse of antibiotics in livestock may cause mutations in this microorganism, thus aiding its spread to other species (4,5). Currently, *S. thoraltensis* is not considered pathogenic to humans. However, it has been found to colonize the oral mucosa of diesel industry workers (6). Furthermore, it has been identified as an etiological agent in cases of chorioamnionitis (7), postpartum pneumonia (8) and fever of unknown origin secondary to bacteremia (9). Recently, *S. thoraltensis* was isolated from the blood samples of an elderly individual with a prosthetic heart valve diagnosed with endocarditis (10). In 2020, the first case of bacterial endocarditis attributable to *S. thoraltensis* emerged in Mexico, affecting an immunocompetent patient with no prior history of heart valve replacement, confirmed via histopathological analysis (11).

Therefore, in line with this emerging pattern, the present study describes the case of a 38-year-old healthy female patient diagnosed with an abscess in the posterior uterine region due to *S. thoraltensis*.

## Case report

**Figure 1:**
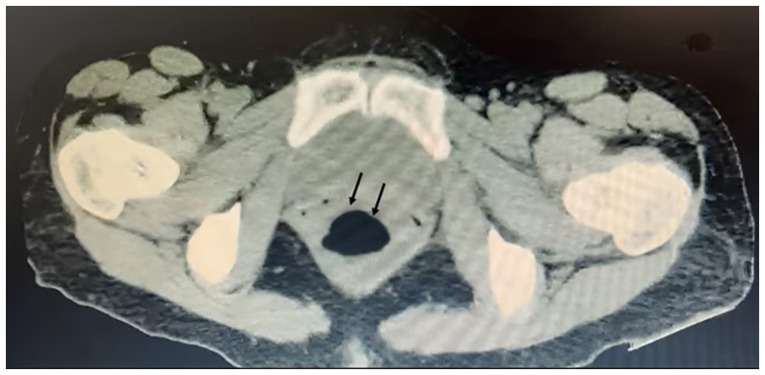
Abdominal computed tomography scan. An hypogenic mass located in the region posterior to the uterus of the patient is shown (black arrows).

A 38-year-old female patient with no prior medical relevant history was admitted to the Emergency Department of the Hospital Regional Universitario of Colima (Colima, Mexico). The individual reported an acute episode of abdominal pain localized in the left iliac fossa, which radiated to the hypogastrium and right iliac fossa over the past 10 days; the pain was intermittent and accompanied by dysuria, vesical tenesmus and constipation persisting for 4 days. Following hospital admission, the patient was transferred to the Gynecology and Obstetrics Department. Following the diagnosis, the patient underwent biochemical and hematological testing. The initial complete blood count revealed low levels of erythrocytes (3.71 million/µl) and hemoglobin (10.30 g/dl). Additionally, leukocytosis was observed (26,500/mm³), predominantly due to neutrophils (86%, with a total of 22,800 neutrophils/mm³). An increase in acute-phase reactants was also noted, with an erythrocyte sedimentation rate of 16 mm/h and C-reactive protein levels of 25 mg/dl. Subsequently, an abdominal tomography scan was requested which revealed a cystic lesion measuring 81x72x67 mm, anteriorly displaced from the uterus, probably due to adnexal origin, with attenuation coefficients of 15 Hounsfield units (HU) and posterior to the rectum (Fig. 1). Evidence of mechanical subocclusion was observed. Thus, surgical management was decided.

An exploratory laparotomy was performed, in which it is not possible to identify the ovarium due to the presence of multiples adherences, whereby adhesiolysis through the blunt technique was executed. In the posterior region of the uterus and sigmoid, an abscess was identified. Therefore, the cavity was drained and 150 cc of purulent material were obtained, which were sent for microbiological culture. Bacteriological analysis using the VITEK 2.0 system^®^ (supplied by BioMérieux Mexico) revealed the presence of *S. thoraltensis* with 97% diagnostic certainty. Treatment was initiated with a triple antibiotic regimen, including doxycycline (100 mg orally every 12 h), metronidazole (500 mg orally every 12 h) and ceftriaxone (1 g daily) for 7 days. At 24 h following the initiation of antimicrobial therapy, follow-up tests were conducted, which revealed a decrease in the erythrocyte sedimentation rate and C-reactive protein levels (5 mm/h and 10 mg/dl, respectively), as well as a reduction in the leukocyte count (14,000/mm³), with 82.8% neutrophils (11,600 neutrophils/mm³). Due to infrastructural limitations, a follow-up computed tomography scan was not performed. However, based on the clinical and paraclinical improvement observed during the follow-up of the patient, the resolution of the clinical condition was established. The symptoms of the patient continued to improve, and following 72 h of treatment, all biochemical and hematological test results were normalized.

## Discussion

The present study describes a concise case report detailing the clinical presentation of a 38-year-old female patient without any notable medical or surgical history, who was diagnosed with a bacterial abscess in the posterior uterine region secondary to *S. thoraltensis* infection. To the best of our knowledge, information about *S. thoraltensis* involvement in human infection is recent and limited.

Recent reports published in 2024 have indicated an increase in the incidence of endocarditis attributed to this etiological agent. In fact, there is a growing number of reports linking endocarditis to the presence of this pathogen. The first case, published by Abid *et al* (12), described a middle-aged male patient with no prior medical history, who presented with acute infectious endocarditis. Upon analysis, the presence of *S. thoraltensis* was confirmed as the causative agent. Notably, the patient had a history of recreational drug use and occupational exposure to livestock (rabbits), which are known reservoirs of *S. thoraltensis* (12). The latest report published in 2024 describes the case of a 65-year-old male patient with multiple comorbidities, including congestive heart failure due to dilated cardiomyopathy, chronic obstructive pulmonary disease, and alcoholic cirrhosis resulting from 20 years of chronic alcoholism, presented with an acute clinical onset. Endocarditis was confirmed both clinically and via ultrasound. The isolated etiological agent was *S. thoraltensis*, after which antibiotic treatment was initiated. However, the clinical condition of the patient deteriorated, ultimately leading to his death due to septic shock (13).

Fortunately, in the case described herein, the patient responded adequality to treatment with doxycycline and metronidazole, similar to a previous report in which the double antibiotic regimen exhibited efficacy (14). It should be mentioned that mammals, particularly quadrupeds, serve as the natural reservoir for this bacterium. Consequently *S. thoraltensis* infection can be classified as a zoonotic disease, with its transmission mechanism plausible associated to food industry (15). Notably, in recent times the number of zoonotic diseases has been increased, encompassing around 75% of all emergent diseases (16). A common strategy employed to diminish the risk of zoonotic diseases is the routine administration of broad-spectrum antibiotics in farms and the food industry. It is estimated that these sectors annually administer thousands of tons of antibiotics (17). On this point, a previous report noted that the excessive use of antibiotics may facilitate the development of pathogenic characteristics in previously innocuous microorganisms (18).

This phenomenon reveals the increasingly evident impact of climate change, which modifies the biochemical interactions between microorganisms and their environment. These changes can disrupt the normal colonization patterns of biological niches and negatively affect the genetic and molecular barriers between host and vectors. It has been theorized that in response to these changes, microorganisms can modify their gene expression to produce unpredictable interactions (19,20). Moreover, the rise in global temperatures accelerates the metabolism of bacteria, although this effect can be mitigated with antibiotics. The selective elimination of pathogenic bacteria could indirectly favor colonization by atypical species, as has been reported for some species of streptococcus (21). Subsequently, if this trend continues, an artificial regulatory effect will be exerted that favors the appearance of emerging pathogens that were previously considered benign.

## Data Availability

The datasets used and/or analyzed during the current study are available from the corresponding author on reasonable request.
